# The metagenomics RAST server – a public resource for the automatic phylogenetic and functional analysis of metagenomes

**DOI:** 10.1186/1471-2105-9-386

**Published:** 2008-09-19

**Authors:** F Meyer, D Paarmann, M D'Souza, R Olson, EM Glass, M Kubal, T Paczian, A Rodriguez, R Stevens, A Wilke, J Wilkening, RA Edwards

**Affiliations:** 1 Mathematics and Computer Science Division, Argonne National Laboratory, 9700 S Cass Avenue, Argonne, IL 60439, USA; 2 Computation Institute, University of Chicago, Chicago, IL 60637, USA; 3 Department of Computer Science, San Diego State University, 5500 Campanile Drive, San Diego, CA 92182, USA

## Abstract

**Background:**

Random community genomes (metagenomes) are now commonly used to study microbes in different environments. Over the past few years, the major challenge associated with metagenomics shifted from generating to analyzing sequences. High-throughput, low-cost next-generation sequencing has provided access to metagenomics to a wide range of researchers.

**Results:**

A high-throughput pipeline has been constructed to provide high-performance computing to all researchers interested in using metagenomics. The pipeline produces automated functional assignments of sequences in the metagenome by comparing both protein and nucleotide databases. Phylogenetic and functional summaries of the metagenomes are generated, and tools for comparative metagenomics are incorporated into the standard views. User access is controlled to ensure data privacy, but the collaborative environment underpinning the service provides a framework for sharing datasets between multiple users. In the metagenomics RAST, all users retain full control of their data, and everything is available for download in a variety of formats.

**Conclusion:**

The open-source metagenomics RAST service provides a new paradigm for the annotation and analysis of metagenomes. With built-in support for multiple data sources and a back end that houses abstract data types, the metagenomics RAST is stable, extensible, and freely available to all researchers. This service has removed one of the primary bottlenecks in metagenome sequence analysis – the availability of high-performance computing for annotating the data.

## Background

The genomic revolution of the 1990s has yielded almost a thousand sequenced microbial genomes. More recently, the explosion of random community genomics, or metagenomics, where DNA is sequenced directly from environmental samples has provided insights into microbial communities. Currently, two approaches to sequencing metagenome samples are commonly used. In the traditional approach, DNA is cloned into BACs, or small plasmids, and dideoxy chain termination sequencing ("Sanger sequencing") is used to determine the sequences [1,2]. In the alternative approach, DNA is sequenced without cloning, using one of the so-called next-generation sequencing techniques, usually pyrosequencing. Both approaches have advantages and disadvantages. For example, Sanger sequencing generates longer sequence reads but has inherent biases due to the cloning. Pyrosequencing has much higher throughput and a lower error rate per base sequenced compared to Sanger sequencing, but those errors are biased toward certain mistakes [3].

Regardless of the sequencing approach used to generate the data, the first steps in analysis of any metagenome involve comparing those sequences to known sequence databases. This computationally intensive task provides the basic data types for many subsequent analyses, including phylogenetic comparisons, functional annotations, binning of sequences, phylogenomic profiling, and metabolic reconstructions.

Here we describe the development of a freely available, fully automated open source system for processing metagenome sequence data to generate these basic elements. A public implementation of this system has been provided for all researchers to analyze their metagenomes. Our service, the metagenomics RAST server (mg-RAST for short), is available over the web to all researchers, and access is not limited to specific groups or data types. Almost 500 metagenomes have been processed through the beta version of the pipeline so far.

## Implementation and results

**Figure 1:**
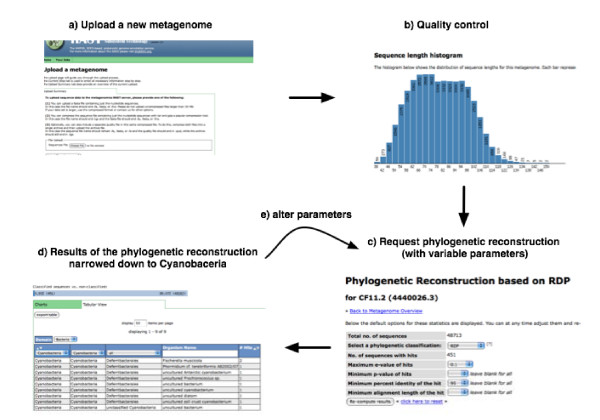
**After uploading a dataset (a), the system computes initial quality control (b) and allows the user to set the parameters for phylogenetic analysis (c).** The system then displays the results (d) and allows the user to alter the parameters (e). Data shown in this example is from the dataset CF11.2 (ID:4440026.3) that is publicly available in the MG-RAST server.

The MG-RAST server is an open source system based on the SEED framework for comparative genomics [4,5]. Users can upload raw sequence data in fasta format; the sequences will be normalized and processed and summaries automatically generated. Genome annotation systems are ever evolving; therefore, in order to accommodate new methods that may be developed, the pipeline was designed with a modular framework that allows the rapid addition of new analysis steps or comparative data at any stage of the analysis. The server provides several methods to access the different data types, including phylogenetic and metabolic reconstructions, and the ability to compare the metabolism and annotations of one or more metagenomes and genomes. In addition, the server offers a comprehensive search capability. Access to the data is password protected, and all data generated by the automated pipeline is available for download and analysis in variety of common formats. Here we describe the key components of the pipeline, which are summarized in Figure 1.

### User Registration and Management

The user registration serves two functions: to limit access to each data set to the user and their colleagues and to secure a valid email address in case correspondence is required, for example if a data-processing problem occurs. Once logged in, users can view their own metagenomes, those to which the owner has granted them rights, and the default set of publicly available metagenomes. The system supports delegation of authorization so that users can allow others to access one or more of their metagenomes. In addition, data owners can release their metagenomes to the public at any point, allowing all users of the system to view their data.

### Data Types

The pipeline accepts data in a number of formats: 454 reads may be uploaded directly in the format delivered by 454 [6], and fasta files typical of Sanger-sequences and used by other platforms may also be uploaded. The pipeline will also accept assembled sequences in fasta format. Sequence data may be compressed by one of several common computer programs to speed upload.

Users may choose to upload raw unassembled reads or assembled contigs. As discussed below, each approach has advantages and disadvantages. Users with a limited number of larger contigs, where the average contig length exceeds 40 kb, should consider using the RAST server for the analysis of complete Bacterial and Archaeal genomes [7].

The Genomics Standards Consortium has proposed a minimal set of data, called the Minimum Information about a Genome Sequence (MIGS) [8], that should be collected with every metagenome sequence. Although this is an evolving standard, the metagenomics-RAST server is MIGS-compliant. Metadata, accessory data about the metagenome (e.g., date and location where the sample was collected), is requested from the user at the time of sequence submission. This data is stored with the user's data and can be provided to the GSC genome catalogue, and other archives, when the sequence data is ready for public release.

### Implementation and Core Analyses

The pipeline is implemented in Perl by using a number of open source components, including the SEED framework [4], NCBI BLAST [9], SQLite, and Sun Grid Engine [10] as components. The system also uses the publicly available SEED subsystems, SEED nr, and FIGfam protein families (see ).

The distinct steps are implemented to provide a flexible, extensible processing pipeline. The steps incrementally add data to a self-contained "job directory" that contains all job-relevant data in flat file and SQLite [11] format. Relational database technology is used to efficiently provide a mapping of sequences in a metagenome to both organisms and metabolic functions and at the same time allow the user to change the parameters for the underlying sequence matches. The user interface enables the download of the user's job directories, and a future version of the software will allow uploading of user-created directories into the server.

**Figure 2:**
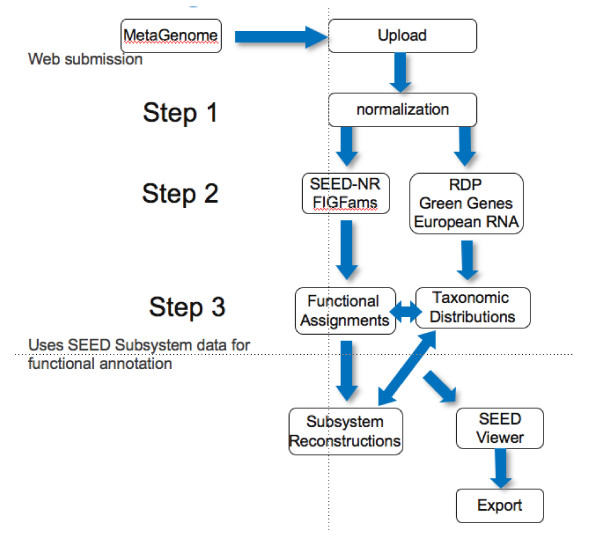
**Overview of the workflow implemented in the metagenomics RAST pipeline.** Three distinct stages of processing are executed, each adding data to a single directory, and ultimately enabling web-based browsing of results.

After uploading the data, a normalization step (see Figure 2) is executed, generating unique internal IDs and removing exactly duplicate sequences from 454 data sets. (These sequences are an artefact of the sequencing technique and are not scientifically meaningful [12].)

In the second step, the sequences are screened for potential protein encoding genes (PEGs) via a BLASTX [9] search against the SEED comprehensive nonredundant database sourced from the INSDC databases, sequencing centers, and other sources [4]. An expect value (E) cut-off of 0.01 is used to pick up potentially coding elements. (This was chosen empirically to increase the number of potentially coding elements while not being overwhelming for data analysis.) In parallel with the BLASTX searches, the sequence data is compared to all accessory databases by using the appropriate algorithms and significance selection criteria. These databases include several rDNA databases, including GREENGENES [13], RDP-II [14], and the European 16S RNA database [15], and boutique databases such as the chloroplast database, mitochondrial database, and ACLAME database of mobile elements [16]. The search criteria are specific for each database. For example, screens for ribosomal RNA genes are performed by using BLASTN against the rDNA databases, but much more stringent selection criteria are used to identify candidate RNA genes than for identifying protein-encoding genes (by default, the similarity must exceed 50 bp in length and have an expect value less than 1 × 10^-5^).

In the third step, these matches to external databases are used to compute the derived data. First, a phylogenomic reconstruction of the sample is computed by using both the phylogenetic information contained in the SEED nr database and the similarities to the ribosomal RNA database. Functional classifications of the PEGs are computed by projecting against SEED FIGfams [17] and subsystems based on these similarity searches [4]. These functional assignments become the raw input to an automatically generated initial metabolic reconstruction of the sample, providing suggestions for metabolic fluxes and flows, reactions, and enzymes.

**Figure 3:**
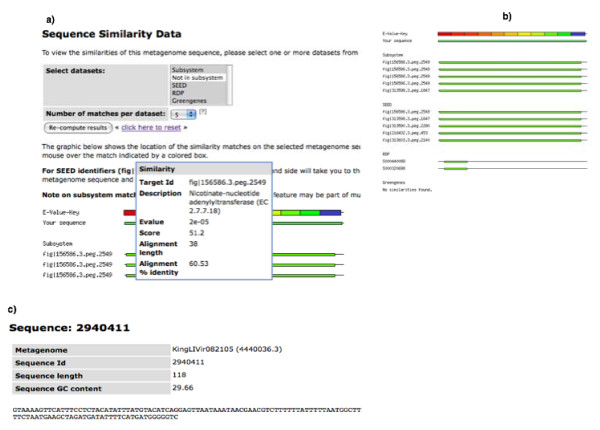
**We emphasize data accessibility, (a) sequence analysis results (e.g. BLAST matches) and all sequences in a metagenome are visible and can be downloaded.** In addition the server provides an overview (b) of the sequence analysis results per fragment in a metagenome (c).

One of the design goals of this server was easy accessibility via a web-based interface. The interface provides views for browsing and analysis of the data, as well as a means to download all result tables and the sequences for every subset displayed. Figure 3 provides an overview of the various elements of the user interface and highlights the options for downloading various subsets. The user interface provides a means to alter some of the parameters used to compute the functional, metabolic, and phylogenetic reconstruction. This allows more stringent match criteria (e.g., expectation value, score, overall percent identity, length of match, and number of mismatches); and, by restricting the matches, the derived data is dynamically changed. The default parameters have been chosen by empirical testing and represent a tradeoff between accuracy and specificity.

### Comparative Metagenomics

The abundance of comparative metagenomics tools is central to the utility of the mg-RAST platform. Various tools have been built into the framework, allowing users to compare their data against other metagenomes or complete genomes taken from the SEED [4] environment. The subsystems heat map and the taxonomic heat map provide comparative metagenomics summaries that encapsulate the differences between samples.

The subsystem comparison tools identify the number of pegs in each metagenome that are connected to a subsystem via protein level similarity. Based on these connections, each subsystem present in a sample is scored by counting the number of sequences that are similar to a protein in each subsystem. This score is divided by the total number of sequences from the sample that are similar to any protein in a subsystem, to give a fraction of sequences in subsystems that are in a given subsystem. This approach allows comparisons between samples that have different numbers of sequences. Since the fractions tend to be small (a few sequences hit each subsystem, but there are now over 600 subsystems in the SEED), the scores can be factored for display purposes. Furthermore, a nonquantitative approach is provided to group the subsystem scores, emphasizing those subsystems that are most different between the samples. Moreover, the display can be limited to specific areas of metabolism, or other subsystem groups, as desired by the user.

The taxonomic heat map works in an analogous fashion but highlights the different taxonomic profiles in each sample, as determined by the phylogenetic or phylogenomic approaches selected by the end user (e.g., 16S comparisons, phylogenomics from BLAST results). Again, samples may be grouped in a nonquantitative fashion to rapidly highlight particular phylogenetic groups that predominate in different samples.

Often a metagenome comprises a few dominant organisms, and many of the pathways in the metagenome can be predicted. The automatically generated metabolic reconstructions can be compared to any given metagenome or complete microbial genome. This approach highlights subsystems that are unique to a metagenome, a comparative genome, or the subsystems common to both. With these tools, users can identify shared metabolism present in their samples.

**Figure 4:**
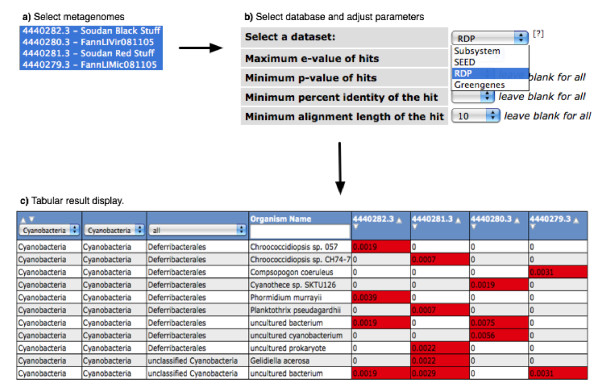
**Comparing the phylogenetic composition of four metagenomes.** Initially (a) the user selects a subset of metagenomes or genomes (here we selected 2 Soudan mine samples and 2 marine samples). The next step (b) allows selecting the basis for the comparison (protein-based-only SEED subsystems or all SEED proteins vs. RNA based RDP or Greengenes) and the parameters for the matches. The parameters include e-value, minimal alignment length, p-value, and percent identity. Finally, the result (c) is displayed in tabular format, in which a heatmap-style color coding is used to highlight differences. The resulting table can be downloaded as a spreadsheet.

## Discussion

A completely new public metagenome annotation system has been developed and released. The process is the result of several years of planning and engineering. Designed to leverage the SEED microbial genome annotation platform, the mg-RAST platform provides seamless integration of metagenome data, microbial genomics, and manually curated annotations. Each metagenome project has its own requirements for stringency, datasets to be analyzed, and output format for results. The metagenomics SEED pipeline was designed to allow alterations to the parameters for the sequence matches underlying both the phylogenetic and metabolic reconstructions to restrict matches. It has been built by using an extensible format allowing the integration of new datasets and algorithms without a need for recomputation of existing results.

The mg-RAST service handles both assembled and unassembled data. Each approach has advantages that should be considered when comparing metagenomes. For example, if one is carrying out comparative metagenomics or if statistics are being used to compare samples [18,19], the sequences cannot be assembled, since the assembly process loses the frequency information critical for determining differences between samples. In contrast, assembled sequences tend to be longer and therefore more likely to accurately identify gene function or phylogenetic source from binning [20].

The analytical methods integrated into the pipeline provide core annotation and analysis tools to compare and contrast a diverse set of metagenomes [21-24]. The approach underlying the subsystems-based functional analysis of metagenomes has been validated with 90 different samples from nine major biomes. The analysis demonstrated that the biomes could clearly be separated by their functional composition [25]. All of the metagenomes present in that study are included in the publicly available datasets visible in the mg-RAST server.

Although the service contains core functionality for the annotation and analysis of metagenomes, many of the techniques traditionally used for genome analysis (e.g., approaches for the prediction of coding sequences) either do not work with metagenomes or show a significant performance degradation [26]. Many of the differences between complete genome annotation and metagenome annotation are reminiscent of those encountered previously with the analysis of expressed sequenced tags [27]. Therefore, new analytical methods are needed to fully understand metagenomics data. The most obvious problem is with the large number of unknown sequences in any sample. Depending on the specific sample processed, as few as 10% of the sequences or as many as 98% of the sequences may have no known similarity to anything in the database [28]. We and others are developing new binning, clustering, and coding region prediction tools to handle these unknown sequences, and effective tools will be incorporated into the pipeline when available. Another problem is that the rapid pace with which sequence data is being generated outpaces increases in computational speed, and therefore improvements in common search algorithms are required to ensure that sequence space can be accurately and efficiently searched. A third problem, common to all annotation platforms, is that metabolic reconstructions and analyses are dependent on the underlying quality of the data. The SEED has the most consistent and accurate microbial genome annotations of any publicly available source because of the subsystems approach to annotation. However, the SEED subsystems are necessarily focused on core metabolism and pathogenesis of a select few organisms. Comprehensive subsystem coverage of secondary metabolism, and especially of metabolism specific to diverse environments, is required to truly comprehend those data sets.

## Conclusion

We have provided a free, public resource for the analysis of metagenome sequence data. Our service does not require a specific type of sequence data and has no requirement for release or control of the data. All sequence data remains protected by a password mechanism and is visible only to permitted users. This metagenomics annotation pipeline was specifically developed to handle pyrosequencing data and accommodate some of the nuances associated with that data. However, the tools and approaches we have developed are applicable and available for any metagenome project, regardless of sequence type. This service has removed one of the primary bottlenecks in metagenome sequence analysis – the availability of high-performance computing for annotating the data.

## Availability

The service is available to all users after a simple registration process. In addition to being available through the integrated SEED-Viewer [29] interface, all results are available for download in variety of formats, including GFF3, GenBank, and flat text formats (e.g., tab-separated text for use in spreadsheets). The server is made available on a best-effort basis, and all underlying data and software are open source (please see ).

Plans include the development of novel tools to allow systematic data mining in the samples and improved support for in-depth analysis of 16S-based metagenome data sets.

## Abbreviations

RAST: Rapid Annotation using Subsystems Technology; mg-RAST: RAST for metagenomes.
